# Robot-Assisted versus Conventional Open Kidney Transplantation: A Meta-Analysis

**DOI:** 10.1155/2020/2358028

**Published:** 2020-12-03

**Authors:** Guangxiang Liu, Yongming Deng, Shenjie Zhang, Tingshen Lin, Hongqian Guo

**Affiliations:** Department of Urology, Nanjing Drum Tower Hospital, The Affiliated Hospital of Nanjing University Medical School, Institute of Urology Nanjing University, Nanjing 210008, China

## Abstract

**Background:**

Perioperative and follow-up outcomes for patients that received robot-assisted kidney transplant (RAKT), compared to patients that received conventional open kidney transplant (OKT), remain unknown. We performed a meta-analysis of controlled studies to compare the safety and efficacy of RAKT versus OKT.

**Methods:**

Systematic searching of PubMed, Embase, and Cochrane Library databases was performed to identify relevant randomized or nonrandomized controlled studies. Perioperative, in-hospital, and follow-up outcomes were summarized. A random-effect model incorporating the potential heterogeneity was used to synthesize the results.

**Results:**

Six nonrandomized controlled studies including 263 patients with RAKT and 804 patients with OKT were included. Pooled results showed that compared to those that received OKT, patients that received RAKT had significant higher rewarming time (mean difference (MD): 20.8 min, *p* < 0.001) and total ischemia time (MD: 17.8 min, *p* = 0.008) but a lower incidence of surgical site infection (SSI, risk ratio (RR): 0.22, *p* = 0.03). The incidence of delayed graft function was comparable between groups (RR: 1.10, *p* = 0.82), and the length of hospital stay was similar (MD: -2.03 days, *p* = 0.21). During a follow-up of 31 months, patients that received RAKT and OKT had similar serum creatinine levels (MD: 10.12 mmol/L, *p* = 0.42) and similar incidences of graft rejection (RR: 1.16, *p* = 0.53), graft failure (RR: 0.94, *p* = 0.79), and all-cause mortality (RR: 1.16, *p* = 0.77).

**Conclusion:**

Current evidence from nonrandomized studies suggests that RAKT is associated with a lower risk of SSI and similar midterm functional and clinical efficacy compared to OKT. Randomized studies are needed to validate these findings.

## 1. Introduction

Kidney transplantation is the final promising treatment option for patients with end-stage renal disease (ESRD) [1, 2]. Since the initial successful case in 1954, conventional open kidney transplant (OKT) surgery with anastomosis of the graft vessels to the recipient's iliac vessels has become the standard procedure [2]. However, OKT has been associated with a higher risk of wound complications [3], particularly in recipients with obesity, diabetes, critical illness, and immunosuppression [4–6]. Moreover, the relatively larger incision of OKT has been recognized as an important cause of surgical site infection (SSI) after the surgery [7]. Accordingly, minimally invasive surgery using laparoscopy has been attempted for kidney transplantation [8]. However, the technical difficulties in performing deep anastomosis in the pelvis limited its clinical application [9]. During the last 20 years, the introduction of the da Vinci robotic surgical system has innovated in the use of robot-assisted kidney transplant (RAKT) [10]. The robotic surgical system could provide a three-dimensional view with magnification options and multiple degrees of freedom, both of which could enable the precise anastomosis performed in the deep pelvis with smaller incisions [11]. However, besides better skin cosmesis which is evident in RAKT than OKT, efficacies of RAKT compared to OKT on intraoperative, in-hospital, and follow-up outcomes in recipients of kidney transplantation remain to be determined [12, 13]. Although some comparative studies comparing RAKT and OKT have been published [14–20], these studies were of limited scale and their results were not consistent. Therefore, we performed a meta-analysis of controlled studies to compare the safety and efficacy of RAKT versus OKT.

## 2. Methods

This systematic review and meta-analysis study was prepared in accordance with the MOOSE [21] and *Cochrane Handbook* [22] guidelines during the study design, implementation, data analysis, and result reporting processes.

### 2.1. Database Searching

PubMed, Embase, and Cochrane Library databases were searched for relevant studies using the term “robot” OR “robotic,” coupled with “renal” OR “kidney” and “transplantation” OR “transplant.” The search was limited to human studies published in the English language. The reference lists of the related original and review articles were also screened manually for potentially relevant studies. The final literature searching was performed on June 29, 2020.

### 2.2. Study Selection

Studies were included if they fulfilled the following criteria: (1) published as a full-length article in English; (2) designed as randomized or nonrandomized controlled studies, without limitations of the sample size and follow-up duration; (3) including patients with ESRD that received RAKT or conventional OKT; and (4) reported at least one of the following outcomes, including intraoperative outcomes (warm ischemia time, cold ischemia time, rewarming time, total ischemia time, blood loss, and incidence of blood transfusion), in-hospital outcomes (delayed graft function, incidence of SSI, and length of hospital stay), and follow-up outcomes (including serum creatinine (SCr) level during final follow-up and risks of graft rejection, graft failure, and all-cause mortality). Warm ischemia time was defined as the time between clamping the donor graft renal artery and placing the graft onto an ice-slushed bath [23]. Cold ischemia time was defined as the time the graft spends on a bench, in ice slush, before introduction into the recipient [23]. Rewarming time indicated the time the graft spends in the recipient before reperfusion while continuously placing in ice slush [23]. Total ischemia time was cold ischemia time plus rewarming time [23]. Delayed graft function refers to the incidence of acute kidney injury in the first week of kidney transplantation which necessitates a dialysis intervention [24]. Definitions of SSI, graft rejection, and graft failure were inconsistent with the diagnostic criteria that were applied in the original studies [3, 25, 26]. Reviews, editorials, preclinical studies, and single-arm studies without an OKT control group were excluded. When duplications of the data were found, the results of the most recent publications with longer follow-up durations were included in the meta-analysis.

### 2.3. Data Extraction and Quality Evaluation

Two independent authors performed literature searching, data extraction, and quality assessment according to the predefined inclusion criteria. Discrepancies were resolved by consensus and discussion with another author. The extracted data included the details regarding study and recipient characteristics, mean body mass index (BMI), donor characteristics, details of immunosuppressive treatments, and follow-up durations. Moreover, characteristics of the donors were also extracted. The quality of randomized controlled studies was evaluated with the Cochrane Risk of Bias Tool [22]. The quality of nonrandomized controlled studies was evaluated with the Newcastle-Ottawa Scale (NOS) [27]. This scale judges the quality of each nonrandomized controlled study regarding three aspects: the selection of the study groups, the comparability of the groups, and the ascertainment of the outcome of interest.

### 2.4. Statistical Analyses

The mean difference (MD) was used as the general measures for the outcomes of continuous variables, while the risk ratio (RR) was used for the categorized variables. The 95% confidence intervals (CI) for MD and RR were also calculated. The heterogeneity among the included studies was detected by the Cochran *Q* test [22, 28] and the *I*^2^ test [29]. An *I*^2^ > 50% indicated significant heterogeneity. A random-effect model was used to pool the results of the included studies because this model was considered to incorporate the potential heterogeneity of the included studies and could therefore retrieve a more generalized outcome [22]. Potential publication bias was assessed by visual inspection of the funnel plot as well as the Egger regression asymmetry test [30]. RevMan (Version 5.1; Cochrane Collaboration, Oxford, UK) software was used for the meta-analysis and statistics.

## 3. Results

### 3.1. Searching Results

**Figure 1:**
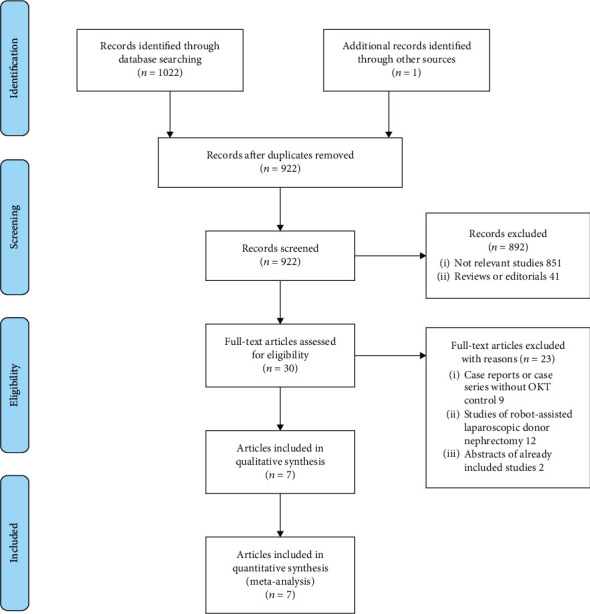
Flowchart of database search and study identification.

The process of literature searching is shown in Figure 1. Briefly, 922 articles were retrieved by initial database searching and exclusion of the duplications. By screening via the title and abstract of the publications, 892 articles were subsequently excluded, mainly because they were irrelevant to the objective of the current study. The remaining 30 articles underwent full-text review, and 23 articles were further excluded because nine studies were case reports or case series of patients with RAKT without OKT control groups, 12 were studies of robot-assisted laparoscopic donor nephrectomy, and the other two were abstracts already included studies. Finally, seven articles [14–20] were retrieved. Since two articles described in-hospital and long-term outcomes of the same study population separately [14, 16], a total of six studies were included.

### 3.2. Study Characteristics and Quality Evaluation

Overall, six nonrandomized controlled studies, including 263 patients with RAKT and 804 patients with OKT, were included in the meta-analysis (Table 1) [14–20]. These studies were published after 2013 and performed in the United States [14–16], Turkey [17], Germany [20], and India [18, 19], respectively. Patients that received RAKT and OKT were generally frequency-matched on age, sex, race, donor compatibility, disease, and dialysis history. The details of immunosuppressive treatments were reported in five of the included studies [14, 15, 17, 19, 20], but not in one study [18] (Table 1). Age, sex, and BMI of the donors are listed in Table 2, while none of the included studies reported the comorbidities of the donors. In five studies, kidney transplant was all performed with living donors [15, 17–20], while in the other study, 93% of the kidney transplant procedure was performed with living donors [14, 16]. The recipients were followed for a mean duration between six and 60 months. The qualities of the included studies were generally good, with the NOS varied between 6 and 8 points (Table 3).

### 3.3. Intraoperative Outcomes

**Figure 2:**
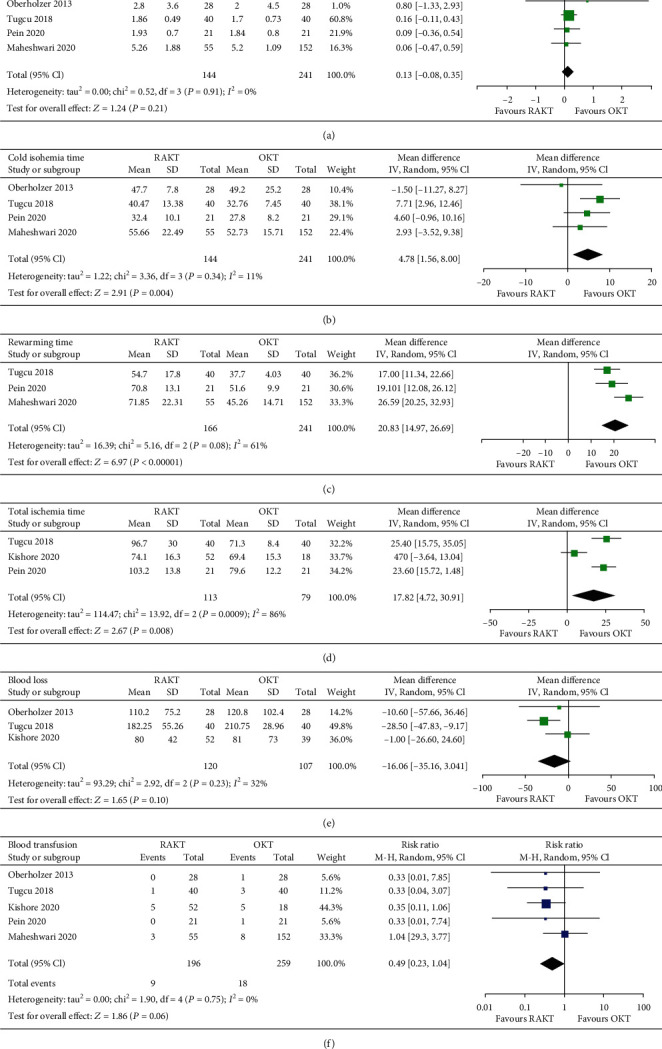
Forest plots for the meta-analysis comparing the influences of RAKT and OKT on intraoperative outcomes: (a) warm ischemia time; (b) cold ischemia time; (c) rewarming time; (d) total ischemia time; (e) volume of blood loss; (f) incidence of blood transfusion.

Pooled results with a random-effect model of four studies [14, 17, 19, 20] showed that the warm ischemia time was not different between patients with RAKT and OKT (MD: 0.13 min, 95% CI: -0.08 to 0.35, *p* = 0.21, and *I*^2^ = 0%; Figure 2(a)). However, RAKT was associated with significantly longer cold ischemia time (four studies [14, 17, 19, 20]; MD: 4.78 min, 95% CI: 1.56 to 8.00, *p* = 0.004, and *I*^2^ = 11%; Figure 2(b)), rewarming time (three studies [17–19]; MD: 20.83 min, 95% CI: 14.97 to 26.69, *p* < 0.001, and *I*^2^ = 61%; Figure 2(c)), and total ischemia time (three studies [17, 18, 20]; MD: 17.82 min, 95% CI: 4.72 to 30.91 min, *p* = 0.008, and *I*^2^ = 86%; Figure 2(d)) compared to OKT. The volume of blood loss (three studies [14, 17, 18]; MD = −16.06 mL, 95% CI: -35.16 to 3.04, *p* = 0.10, and *I*^2^ = 32%; Figure 2(e)) and the incidence of blood transfusion (five studies [14, 17–20]; RR: 0.49, 95% CI: 0.23 to 1.04, *p* = 0.06, and *I*^2^ = 0%; Figure 2(f)) were not statistically different between patients that were treated with RAKT and OKT.

### 3.4. In-Hospital Outcomes

**Figure 3:**
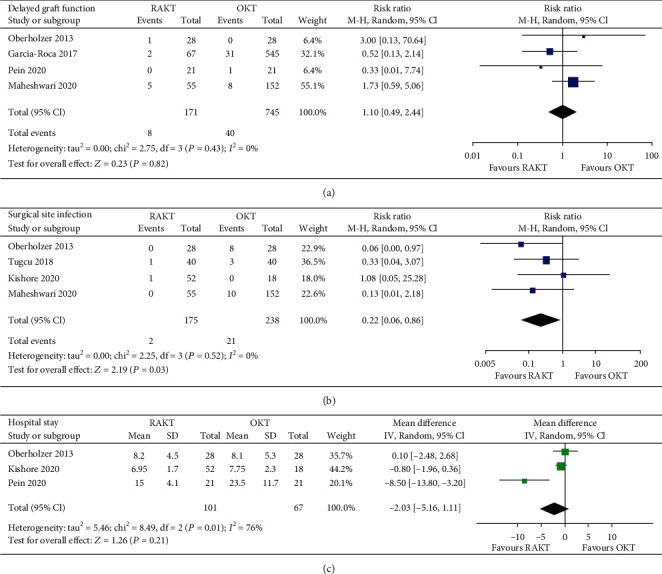
Forest plots for the meta-analysis comparing the influences of RAKT and OKT on in-hospital outcomes: (a) incidence of delayed graft function; (b) incidence of SSI; (c) lengths of hospital stay.

The incidence of delayed graft function was not significantly different between patients in the RAKT and OKT groups (four studies [14, 15, 19, 20]; RR: 1.10, 95% CI: 0.49 to 2.44, *p* = 0.82, and *I*^2^ = 0%; Figure 3(a)). However, RAKT was associated with a significantly lower risk of SSI compared to OKT (four studies [14, 17–19]; RR: 0.22, 95% CI: 0.06 to 0.86, *p* = 0.03, and *I*^2^ = 0%; Figure 3(b)). The length of hospital stay was not different between patients that were treated with RAKT and OKT (three studies [14, 18, 20]; MD: -2.03 days, 95% CI: -5.16 to 1.11, *p* = 0.21, and *I*^2^ = 76%; Figure 3(c)). The incidence of urological complications was reported in only one study [15]. One patient receiving OKT had a urological complication in this study [15], while not for the patients receiving RAKT.

### 3.5. Follow-Up Outcomes

**Figure 4:**
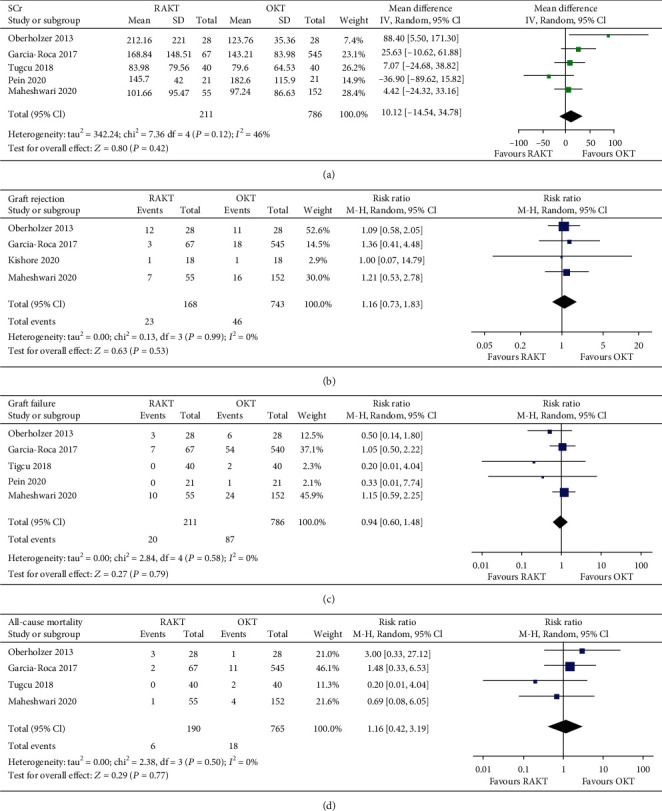
Forest plots for the meta-analysis comparing the influences of RAKT and OKT on follow-up outcomes: (a) SCr levels at final follow-up; (b) incidence of graft rejection; (c) incidence of graft failure; (d) incidence of all-cause mortality.

During a mean follow-up of 31 months (6 to 60 months), SCr levels in patients that received RAKT and OKT were not significantly different (five studies [14, 15, 17, 19, 20]; MD: 10.12 mmol/L, 95% CI: -14.54 to 34.78, *p* = 0.42, and *I*^2^ = 46%; Figure 4(a)). Moreover, patients that received RAKT and OKT had similar incidences of graft rejection (four studies [14, 15, 18, 19]; RR: 1.16, 95% CI: 0.73 to1.83, *p* = 0.53, and *I*^2^ = 0%; Figure 4(b)), graft failure (five studies [14, 15, 17, 19, 20]; RR: 0.94, 95% CI: 0.60 to 1.48, *p* = 0.79, and *I*^2^ = 0%; Figure 4(c)), and all-cause mortality (four studies [14, 15, 17, 19]; RR: 1.16, 95% CI: 0.42 to 3.19, *p* = 0.77, and *I*^2^ = 0%; Figure 4(d)).

### 3.6. Publication Bias

The publication bias for the current meta-analysis was unable to estimate since only three to five studies were available for each outcome.

## 4. Discussion

In this meta-analysis of nonrandomized controlled studies, we found that although RAKT was associated with longer cold ischemia time, rewarming time, and total ischemia time compared to conventional OKT, the volume of blood loss and incidence of blood transfusion were not statistically significant between patients of the two groups. Moreover, patients that received RAKT had a lower incidence of SSI, while the risk of delayed graft function and the length of hospital stay were not significantly different. As for the midterm clinical outcomes, SCr levels at final follow-up were not significantly different for patients that were treated with RAKT and OKT, and the risks of graft rejection, graft failure, and all-cause mortality were similar between patients in both groups. Taken together, current evidence from nonrandomized studies suggests that RAKT may be associated with a lower risk of SSI and similar midterm functional and clinical efficacy compared to OKT. Randomized studies are needed to validate these findings.

To the best of our knowledge, our study is the first meta-analysis summarizing the efficacy and safety of RAKT compared to OKT in recipients with ESRD. Although the promising efficacy of RAKT in these patients has been reported in previous studies, most of them were case reports or case series without a control group of OKT [12, 31–33]; the influences of RAKT on short-term and follow-up outcomes in kidney transplant recipients as compared with OKT remain undetermined. By pooling the available controlled studies, our study showed that compared to OKT, RAKT was associated with longer rewarming time and total ischemia time. The reasons, from our perspective, may be accounted for by the lack of initial experience of the surgeon. In RAKT, additional time may be needed to close the insertion site, manipulate the graft kidney, and apply vascular occlusion clamps, all of which could lead to the extension of rewarming time and total ischemia time [34]. With the accumulating of cases performed, rewarming time and total ischemia time could be shortened for an experienced surgeon [34]. Another important finding regarding the short-term outcome is that the incidence of SSI was significantly reduced in patients treated with RAKT compared to those treated with OKT. This is particularly important for obese patients who were previously less likely to receive kidney transplantation due to a higher incidence of wound infection and overall poor prognosis [35]. This may be partially attributed to the smaller incision in RAKT. Besides, replacing the suprainguinal incision in a highly colonized area in OKT with a periumbilical incision in RAKT may also be responsible for the resulting lower incidence of SSI. As for the functional outcome, the incidence of delayed graft function and the level of SCr during follow-up up to five years were similar between patients treated with RAKT and OKT, suggesting that the mild difference in rewarming time and total ischemia time may not significantly affect the graft function. More importantly, we found that the midterm incidences of graft rejection, graft failure, and all-cause mortality were similar between groups, which further confirmed that RAKT is safe and effective in ESRD patients as conventional OKT. These findings highlight the rationale to perform a randomized clinical trial to validate the safety and efficacy of RAKT.

Some limitations of this meta-analysis should be mentioned. Firstly, from a clinical perspective, the potential benefits of RAKT on accurate vascular anastomosis are the most important outcome that the kidney transplantation surgeons would like to know. However, since none of the included studies compared this outcome directly, it remains unknown whether RAKT compared to OKT is associated with any benefit on the accurate vascular anastomosis. Furthermore, the potential benefits of RAKT largely depend on the experiences and skills of this novel technique. Therefore, at the current stage, it may be too early to recommend RAKT in real-world clinical practice. Besides, only nonrandomized controlled studies were identified. Although these studies included patients in RAKT and OKT who had been balanced for most study characteristics, the results were based on univariate analysis. We could not exclude the possibility that differences in some residual study characteristics may confound the results, such as the comorbidities of the patients. In addition, studies available for the meta-analysis are limited. We were unable to evaluate the potential influences of patient or study characteristics on the efficacy outcome between groups in a subgroup analysis. Moreover, combining the results of these small-scale studies may remain statistically inadequate to detect potential differences in clinical outcomes between groups. Finally, the mean follow-up duration was 31 months; the long-term efficacy of RAKT compared to OKT remains to be determined.

In conclusion, the results of the meta-analysis showed that RAKT may be associated with a lower risk of SSI and similar midterm functional and clinical efficacy compared to OKT for ESRD patients. Randomized studies are warranted to validate these findings and determine the potential long-term safety and efficacy of RAKT in these patients.

## Figures and Tables

**Table 1:** Characteristics of the recipients.

Study	Country	Design	Methods for group pairing	Number of patients		Mean age (years)		Male (%)		BMI (kg/m^2^)		IS treatments	Follow-up duration (months)
				RAKT	OKT	RAKT	OKT	RAKT	OKT	RAKT	OKT		
Oberholzer 2013	USA	NRCT	Matched pair	28	28	48	50	46	39	43	38	Both induction and maintenance IS applied according to the risk of the patients	60
Garcia-Roca 2017	USA	NRCT	Matched pair	67	545	46	48	48	52	NR	NR	Induction included steroids in all RAKT patients and 73.8% of OKT patients; maintenance IS mostly based on a calcineurin inhibitor in combination with an antimetabolite	36
Tugcu 2018	Turkey	NRCT	Matched pair	40	40	38	42	38	30	23	25	Initiated with antithymocyte globulin, maintenance treatment consisted of prednisone, tacrolimus, and mycophenolate mofetil	6
Kishore 2020	India	NRCT	Matched pair	52	18	39	35	73	77	26	25	NR	20
Pein 2020	Germany	NRCT	Matched pair	21	21	48	45	76	48	26	26	Both induction and maintenance IS applied according to the risk of the patients	13
Maheshwari 2020	India	NRCT	Matched pair	55	152	41	43	76	80	26	24	Induction with antithymocyte globulin or basiliximab, maintenance IS not reported	26

USA: United States of America; BMI: body mass index; RAKT: robot-assisted kidney transplant; OKT: open kidney transplant; NRCT: nonrandomized controlled trials; NR: not reported; IS: immunosuppressing treatments.

**Table 2:** Characteristics of donors of the included studies.

Study	Number of donors		Living donor (%)		Related donor (%)		Mean age (years)		Male (%)		BMI (kg/m^2^)	
	RAKT	OKT	RAKT	OKT	RAKT	OKT	RAKT	OKT	RAKT	OKT	RAKT	OKT
Oberholzer 2013	28	28	93	93	77	65	32	34	57	35	29	31
Garcia-Roca 2017	67	545	100	100	NR	NR	36	42	45	36	30	28
Tugcu 2018	40	40	100	100	NR	NR	NR	NR	NR	NR	NR	NR
Kishore 2020	52	18	100	100	NR	NR	NR	NR	NR	NR	NR	NR
Pein 2020	21	21	100	100	NR	NR	53	54	57	62	27	26
Maheshwari 2020	55	152	100	100	NR	NR	NR	NR	NR	NR	NR	NR

BMI: body mass index; RAKT: robot-assisted kidney transplant; OKT: open kidney transplant; NR: not reported.

**Table 3:** Characteristics of the included studies.

Study	Representativeness of the exposed cohort	Selection of the nonexposed cohort	Ascertainment of exposure	Demonstration that the outcome of interest was not present at the start of the study	Comparability-age and gender	Comparability-other factors	Assessment of outcome	Was follow-up long enough for outcomes to occur	Adequacy of follow-up of cohorts	Total
Oberholzer 2013	0	0	1	1	1	0	1	1	1	6
Garcia-Roca 2017	0	0	1	1	1	0	1	1	1	6
Tugcu 2018	1	1	1	1	1	0	1	0	1	7
Kishore 2020	0	1	1	1	1	1	1	0	1	7
Pein 2020	1	1	1	1	0	0	1	0	1	6
Maheshwari 2020	1	1	1	1	1	0	1	0	1	7

## Abbreviations

ESRD: End-stage renal disease
OKT: Open kidney transplant
SSI: Surgical site infection
RAKT: Robot-assisted kidney transplant
SCr: Serum creatinine
BMI: Body mass index
NOS: Newcastle-Ottawa Scale
MD: Mean difference
RR: Risk ratio
CI: Confidence interval.
